# Veterinary Perspectives on Hemoglobin-Based Oxygen Carriers in Experimental Hemorrhagic Shock: Insights from Rabbit Models

**DOI:** 10.3390/vetsci12050485

**Published:** 2025-05-16

**Authors:** Ștefania-Mădălina Dandea, Alina-Diana Hașaș, Vlad-Alexandru Toma, Maria Lehene, Florina Scurtu, Cosmin Petru Peștean, Răzvan-Andrei Codea, Lucia-Victoria Bel, Iulia Melega, Radu Silaghi-Dumitrescu, Bogdan Sevastre

**Affiliations:** 1 Faculty of Veterinary Medicine, University of Agricultural Science and Veterinary Medicine, 400372 Cluj-Napoca, Romania; alina.hasas@usamvcluj.ro (A.-D.H.); cosmin.pestean@usamvcluj.ro (C.P.P.); razvan.codea@usamvcluj.ro (R.-A.C.); lucia.bel@usamvcluj.ro (L.-V.B.); iulia.melega@usamvcluj.ro (I.M.); bogdan.sevastre@usamvcluj.ro (B.S.); 2 Department of Molecular Biology and Biotechnology, Babeș-Bolyai University, 400084 Cluj-Napoca, Romania; vlad.toma@ubbcluj.ro; 3 Department of Experimental Biology and Biochemistry, Institute of Biological Research from Cluj-Napoca, Branch of National Institute of Research and Development for Biological Sciences Bucharest, 400015 Cluj-Napoca, Romania; 4 Center for Systems Biology, Biodiversity and Bioresources “3B”, Babeș-Bolyai University, 400084 Cluj-Napoca, Romania; 5 Faculty of Chemistry and Chemical Engineering, Babeș-Bolyai University, 400084 Cluj-Napoca, Romania; maria.lehene@ubbcluj.ro (M.L.); violeta.scurtu@ubbcluj.ro (F.S.); radu.silaghi@ubbcluj.ro (R.S.-D.)

**Keywords:** polymerized hemoglobin, glutaraldehyde, oxygen carrier, hemorrhagic shock

## Abstract

Hemoglobin-based oxygen carriers (HBOCs) represent a promising alternative to traditional blood transfusions, offering the advantages of extended shelf life and avoiding blood compatibility limitations and infection risks. Positive effects of hemoglobin-based oxygen carriers (HBOCs) on hemorrhagic shock have been researched across various animal species, including swine, rats, rabbits, guinea pigs, and dogs. As previously described, HBOCs based on ovine hemoglobin display better efficiency in the context of hemorrhagic shock compared to those based on the more commonly used bovine hemoglobin. This was evidenced through higher survival rates and more favorable histopathological and immunological outcomes. The vascular effects of ovine hemoglobin polymerized with glutaraldehyde exposure included the absence of hypertension, minimal endothelial damage with slight alterations in inducible nitric oxide synthase (iNOS), and reduced vascular inflammation mediated by interleukin-10 (IL-10). Ovine hemoglobin has emerged as a particularly promising raw material for the development of HBOCs, surpassing bovine and human hemoglobin due to its advantages in availability and efficacy. Furthermore, reducing oxidative stress by polymerizing hemoglobin with glutaraldehyde is most effective with ovine hemoglobin compared to bovine hemoglobin. This study evaluates the effectiveness of ovine hemoglobin polymerized with glutaraldehyde in managing hemorrhagic shock in rabbits, with a focus on its ability to maintain blood pressure, support oxygen transport, and assess potential systemic and oxidative responses. Fifteen adult New Zealand white rabbits, divided into three equal groups, were included in this study: a negative control group transfused with colloid solutions, a positive control group treated with autotransfusion, and a group receiving HBOCs. All groups underwent a hemorrhagic shock protocol, with 40% of their total blood volume withdrawn under deep anesthesia, followed by transfusions 30 min later. Vital parameters, including invasive arterial blood pressure, heart rate, and end-tidal CO_2_, were measured throughout the experimental procedures. Arterial blood gas samples were collected before the procedures, after hemorrhagic shock induction, and at the conclusion of the transfusion. In summary, HBOCs offer a promising solution for oxygen delivery, but their effects on blood chemistry, particularly CO_2_ and lactate levels, must be considered. Although no direct oxygenation issues were observed in experimental models, elevated CO_2_ levels and the interference of HBOCs with lactate measurements emphasize the importance of vigilant clinical monitoring. Polymerized hemoglobin provides a non-nephrotoxic alternative, but challenges persist in preventing nitric oxide scavenging and ensuring effective oxygen delivery.

## Simple Summary

Hemoglobin-based oxygen carriers (HBOCs) are studied as a promising alternative to traditional blood transfusions, offering longer shelf life and eliminating the need for blood compatibility and infection risk management in both human and veterinary medicine. Among these, ovine hemoglobin polymerized with glutaraldehyde has shown superior outcomes compared to bovine or human hemoglobin, particularly in hemorrhagic shock. It demonstrated better survival rates, minimal endothelial damage, no hypertension, and reduced vascular inflammation via IL-10. This study evaluated ovine HBOC efficacy in 15 New Zealand white rabbits subjected to hemorrhagic shock, divided into three groups: colloid solution (negative control), autotransfusion (positive control), and HBOC treatment. Each rabbit had 40% of its blood volume withdrawn under anesthesia, followed by transfusion 30 min later. Vital signs and arterial blood gases were monitored throughout. The results confirmed that HBOCs maintained blood pressure and oxygen transport, although elevated CO_2_ levels and interference with lactate readings were noted. Despite no direct oxygenation impairment, these findings underscore the need for careful clinical monitoring. Overall, ovine polymerized hemoglobin offers a non-nephrotoxic, effective HBOC solution, though challenges regarding clinical efficiency and accurate biochemical long-term monitoring remain.

## 1. Introduction

Hemoglobin-based oxygen carriers (HBOCs) present a promising solution to the limited availability of blood products in veterinary medicine. In addition, they offer extended shelf life and eliminate the need for cross-matching [1]. Ovine hemoglobin has emerged as a particularly promising raw material for the development of HBOCs, surpassing bovine and human hemoglobin due to its advantages in both availability and efficacy. This is particularly significant given the current limitations in the supply of human hemoglobin and the challenges associated with large-scale recombinant production. Furthermore, reducing oxidative stress by copolymerizing hemoglobin with serum albumin has been shown to be effective, particularly when the albumin is sourced from the same species as the hemoglobin [2].

Positive effects of hemoglobin-based oxygen carriers (HBOCs) on hemorrhagic shock have been demonstrated across various animal species, including swine [3], rats [4], rabbits [5], guinea pigs [6], and dogs [7]. These studies have shown that HBOCs can improve hemodynamic stability, enhance oxygen delivery, and reduce inflammatory responses during resuscitation from hemorrhagic shock

In previous studies involving rats, exposure to ovine polymerized hemoglobin with glutaraldehyde resulted in a reduction in proinflammatory cytokines and matrix metalloproteinases, along with an increase in IL-10. Despite elevated oxidative stress, ultrastructural analysis revealed only minor endothelial damage in the hemorrhage group, and polymerized hemoglobin did not cause significant harm to the vascular endothelium. Key vascular effects of polymerized hemoglobin exposure included the absence of hypertension, minimal endothelial damage with slight alterations in iNOS, and a reduction in vascular inflammation mediated by IL-10 [4].

Additionally, previous research has demonstrated the efficiency of various forms of HBOCs in rabbits. Hemoglobin vesicles (HbVs) in a rabbit model effectively stabilized hemodynamics and improved survival rates, suggesting their potential as a resuscitative agent in hemorrhagic conditions [8]. A novel cross-linked hemoglobin-based oxygen carrier, in rabbits and goats, resulted in improved oxygen delivery, and efficiently managed aspects relevant to hemorrhagic shock scenarios [9]

Polymerized hemoglobin showed efficacy in short-term stabilization, supporting its potential use as a bridge therapy in acute blood loss scenarios [10].

Hemorrhagic shock is a critical condition characterized by a reduction in intra-vascular blood volume to the point where tissue oxygen demand cannot be met, leading to anaerobic metabolism [11]. Critical vital parameters include temperature, heart rate, respiratory rate, end-expiratory carbon dioxide concentration, oxygen saturation, mean arterial pressure (MAP), and systolic and diastolic blood pressure, while paraclinical values encompass hematocrit, blood pH, arterial blood gases, lactate levels, systemic base excess, and coagulation factors [12]. Animal models are essential for understanding the pathophysiology of hemorrhagic shock, with increasing sophistication aimed at mimicking hemostatic and physiological responses [13]. Despite advancements, challenges remain in fully replicating natural conditions and addressing the multifaceted aspects of traumatic hemorrhagic shock in clinical settings [13]. Arterial catheterization, commonly via the carotid artery, facilitates continuous monitoring of mean arterial pressure (MAP) during hemorrhage induction [12].

Compared to traditional blood products, HBOCs are pathogen-free, universally compatible, and do not require cross-matching [14]. Furthermore, in our previous studies comparing different HBOC molecules in rats [15], PEGylated HBOCs improved molecular stability, reduced renal clearance, and extended intravascular half-life, thus enhancing their resuscitative efficiency in hypovolemic conditions. These properties make HBOCs promising candidates for use in emergency medicine and veterinary care.

In human and veterinary medicine, blood and blood product transfusion services are difficult and demanding, considering voluntary donor participation and comprehensive pretransfusional screening protocols. Another factor is the implementation of temperature-controlled systems to ensure the integrity and safety of blood products throughout collection, storage, and distribution [16].

The objective of the present study is to assess the efficacy of ovine hemoglobin polymerized with glutaraldehyde in managing hemorrhagic shock in rabbits, with a focus on its ability to maintain blood pressure and support oxygen transport.

## 2. Materials and Methods

### 2.1. Experimental Animals

This study was conducted on 15 healthy female New Zealand white rabbits, weighing 4.12 ± 0.23 kg, purchased from “Cantacuzino” National Medical-Military Research-Development Institute, located at Splaiul IndependențeiSector 5, București, Romania. which were fed a standard diet. After acclimatization, all of the animals were subject to the same procedure of general anesthesia and hemorrhagic shock induction protocol by extracting 40% of blood within one hour. Then, the animals were divided into three equal groups (five animals in each); the first group received Dextran 70 (60 g/L in sodium chloride 9 g/L) (INFOMED FLUIDS S.R.L., Bucharest, Romania), and the second group received previously collected blood on CPDA (Citrate Phosphate Dextrose Adenine) from Demotek 250 mL blood bags. The third group received glutaraldehyde-polymerized ovine hemoglobin (GPOH). The experiment was conducted in the Laboratory Animal Establishment of the University of Agricultural Science and Veterinary Medicine Cluj-Napoca (Authorization No. 936/06.10.2021), from January 2022 to April 2022.

### 2.2. Bioethics

The experimental protocol followed the PREPARE guidelines; additionally, this study complies with Directive 63/2010/EU. According to Romanian National Law 43/2014, this project was approved by the Institutional Ethics Committee (Decision no. 290/22.11.2021) and authorized by the Cluj Sanitary-Veterinary and Food Safety Department (Authorization no. 284/21.12.2021). All procedures were performed by highly trained professionals, on unconscious animals, under anesthesia; therefore, the severity was classified as non-recovery.

### 2.3. Ovine Blood Collection

Hemoglobin was collected from live, healthy donor rams weighing over 50 kg, with no prior medical history. The animals were housed in the university’s scientific animal facility, monitored daily, and managed according to institutional and national bioethical standards for animal care and welfare. Whole blood was obtained from the jugular vein using a closed collection system, with each sheep donating approximately 250 mL of blood—ensuring that withdrawal did not exceed 10% of total blood volume—into CPDA-1 (Citrate Phosphate Dextrose Adenine) bags (Demotek^®,^ Ilfov, Romania), following mild analgosedation. Blood collection followed a standardized protocol involving light sedation with xylazine (0.1–0.2 mg/kg IM), antisepsis of the jugular area using iodine solution, insertion of a sterile catheter, and a strict aseptic technique with sterile gloves and materials. Sterility was further ensured by using a closed gravity-assisted collection system and disposable sterile components, with the entire procedure conducted by trained personnel in a controlled environment. The blood collection, transportation, and processing of the experimental substances were all carried out on the same day to ensure optimal sample integrity and consistency.

### 2.4. HBOC

Ovine hemoglobin was purified as previously described (6); polymerized hemoglobin was obtained by mixing 1 mM ovine hemoglobin with 9 mM glutaraldehyde (from Sigma-Aldrich, Hamburg, Germany) in PBS. The reaction was left under stirring conditions for 2 h at 4 °C. A four-fold molar excess (with respect to glutaraldehyde concentration) of sodium borohydride was added to the reaction and allowed to react for 15 min in order to reduce the Schiff bases formed and the excess glutaraldehyde. Endotoxin removal was performed using a Pierce High-Capacity Endotoxin Removal Resin.

### 2.5. Anesthesia and Preoperative Care

To ensure adequate analgesia, all animals received intramuscular Buprenorphine (Bupaq 0.3 mg/mL Richter Pharma, Bucharest, Romania) at a dose of 0.04 mg/kg and subcutaneous Meloxicam (Meloxidolor 20 mg/mL Le Vet Farma, Bucharest, Romania) at a dose of 0.5 mg/kg followed by preoxygenation. Anesthesia induction was carried out by the intramuscular administration of Dexmedetomidine (Sedadex, Baia Mare, Romania 0.5 mg/mL) at a dose of 0.08 mg/kg and ketamine (Ketamidor 100 mg/mL Plurivet, Baia Mare, Romania) at a dose of 40 mg/kg. Anesthesia maintenance was achieved using 2% isoflurane, and the rabbits were intubated with endotracheal tubes ranging from 2 to 3.5 mm in diameter. A rigid endoscope with a bright light source guided the intubation process. Following anesthesia, the rabbits were prepared for further procedures. This included oxygenation via ET tube and preparing the auricular regions by shaving them to allow for vascular access to the auricular veins. The ventral region of the neck up to the sternum manubrium was also shaved to facilitate surgical access. Additionally, hair on limb extremities was trimmed, ECG electrodes were placed, and experimental animals were positioned dorsally, and secured in the chosen position using adhesive tape. To ensure venous access, the left lateral auricular vein and left cephalic vein were catheterized using a 22G B. Braun, peripheral venous catheter. This catheterization procedure allowed us to obtain reliable and convenient venous access for fluid or drug administration throughout the anesthesia process. After intubation, all previously shaved regions underwent careful antisepsis using alcohol and chlorhexidine solutions to minimize the risk of infection.

Continuous infusion of ketamine at a rate of 2 mg/kg/h with a constant-rate syringe infusion pump was connected using an Eickemeyer InjectoVET Easy II, Tuttlingen, Germany. This infusion aimed to provide a controlled and desired infusion rate during the experimental protocol. The calculated ketamine volume was diluted in a 10 mL syringe and administered at a rate of 5 mL/h intravenously.

Physiological alterations, including increases in hemodynamic parameters, are anticipated during both the induction and progression of hemorrhagic shock, and were taken into account during data interpretation, in alignment with previously published studies on anesthetic protocols in rabbit models of hemorrhagic shock. To minimize anesthesia- and procedure-related stress, the protocol was designed to optimize vascular stability during surgical interventions and ensure continuous analgesia throughout the experiment [17,18,19,20]. Once intubated, the rabbits were connected to a Mindray PM9000 VETMONITOR for monitoring heart rate, respiratory rate, and oxygen saturation (SPO_2_). The SPO_2_ sensor was placed either on the ear or paw. All animals were spontaneously ventilating and end-tidal CO_2_ levels were measured using a sidestream CO_2_ module connected to the monitor.

Rabbit temperature was monitored by placing an esophageal tube and displayed on the main monitor. All individuals in the considered sample were placed on a BEURER HK123 electrical pad to preserve physiological body temperature throughout the procedures and avoid hypothermia vasoconstriction.

### 2.6. Induction of Hemorrhagic Shock

**Figure 1:**
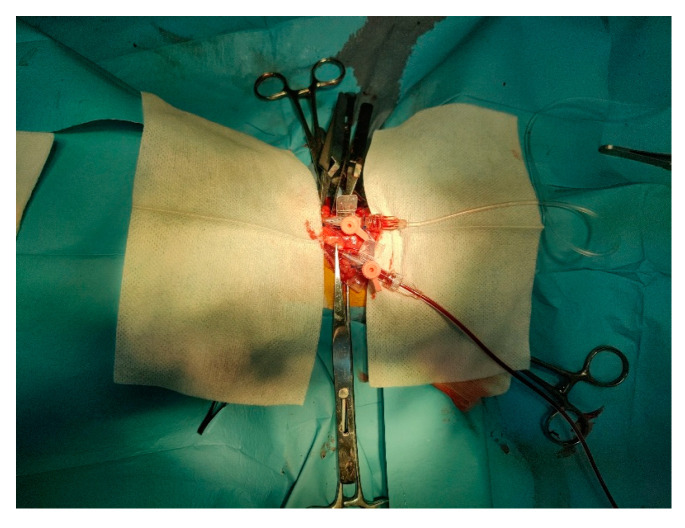
Catheterization of carotid arteries.

All rabbits underwent controlled induction of controlled hemorrhagic shock. A set of ophthalmologic surgical instruments was prepared, providing appropriately sized tools for precise incisions in the cervical region. After preoperative procedures, and ensuring antisepsis, an incision approximately 3 cm in length was made along the ventral midline of the neck to expose the underlying tissues. Cervical muscles and surrounding anatomical structures were dissected to expose both carotid arteries. Desmarres or Knapp retractors were used to retract cervical muscles and surrounding anatomical structures for better exposure and visualization of the procedural area. Dorsal exposure of the carotid arteries was achieved using subcutaneous sutures (3-0) secured with Adson hemostatic forceps (Figure 1).

The right carotid artery was catheterized with a sterile 22G B. Braun peripheral venous catheter and connected to an invasive blood pressure monitoring transducer to provide accurate data on mean arterial pressure (MAP), systolic pressure, and diastolic pressure. The left carotid artery was catheterized with another 22G venous catheter to serve as the bleeding site

The catheter inserted into the left carotid artery was connected to a transfusion tube leading to a Demotek 250 mL transfusion bag. Hemorrhagic shock was induced by withdrawing blood at a rate of 3 mL/kg/min through the carotid artery. This state is achieved when approximately 40% of the total blood volume is removed. The total blood volume is estimated at 8% of the rabbit’s body weight and was calculated accordingly after weighing. Using this figure, the volume equivalent to 40% of the total blood was determined for extraction to induce hemorrhagic shock in 60 min. To maintain constant and controlled bleeding, the bleeding tube was connected to an Eickemeyer infusion pump, InfusoVet II, with the tubing inverted. This configuration ensured controlled exsanguination over 60 min.

The total blood extraction volume was set as the infusion volume on the machine, with the same infusion rate. After stopping bleeding, a 30 min monitoring period allowed us to observe the evolution of hemorrhagic shock signs. After the 30 min rest period, the same volume of experimental substances was subsequently infused to observe the progression and assess the physiological response using colloidal solutions, extracted blood, or HBOC. The infusion was carried out over 60 min. At the end of this study, the animals were killed by isoflurane overdose. The animals were considered deceased when no heart activity was recorded for 5 min time, according to ethical guidelines and protocols.

### 2.7. Blood Monitoring and Vitals

The total duration of the experiment was 180 min, during which vital signs and reflexes were continuously monitored. Monitoring included parameters such as heart rate, respiratory rate, SPO_2_, ETCO_2_, systolic blood pressure, diastolic blood pressure, mean arterial pressure, and temperature through an esophageal tube, using the present monitor. All monitored aspects were observed at different stages of the procedure: After 30 min, which involved surgical procedures and arterial catheterization, the bleeding time began, noted as T1, then at every 15 min during the bleeding time, denoted as T2 (45 min from the start of the experiment), T3 (60 min), T4 (75 min), and T5 (90 min). At T5, bleeding was stopped, and additional data were observed after 30 min at T6 when isovolemic transfusions commenced. Subsequently, data were collected every 30 min during isovolemic transfusion, noted as T7 and T8.

Arterial blood gas analyses were collected and assessed in pre-hemorrhagic, post-hemorrhagic, and post-transfusion stages. Arterial blood was collected from the cannulated carotids into a preheparinized syringe with sodium heparin and immediately connected for evaluation using a Stat Profile Prime Plus device. The results included the following parameters: lactate, creatinine, BUN, bilirubin, total CO_2_, BE ecf, osmolality, and anion gap.

### 2.8. Statistics

The data were expressed as the mean ± Standard Deviation. The normal distribution of the data was assessed using the Shapiro–Wilk test; as the data were normally distributed, statistical analysis among experimental groups was conducted using two-way ANOVA, followed by Bonferroni correction, *p* < 0.05, which was considered significant. Statistical data were generated by using GraphPad Prism 5.0 for Windows, GraphPad Software, San Diego, CA, USA.

## 3. Results and Discussion

### 3.1. Vitals During Experimental Procedures

The heart rate (HR) and respiratory rate (RR) remained within their reference ranges throughout this study. In the HBOC group, during the isovolemic transfusion phase (T7 and T8), HR increased to 232 ± 14.23 bpm and subsequently to 239 ± 9.41 bpm, respectively. Initially, RR was slightly reduced but later increased across all groups; however, these changes were not statistically significant. Core body temperature was initially at the lower end of the physiological range, and further decreased into hypothermia as a result of hypovolemic shock induction. Notably, both blood transfusion and HBOC successfully restored core temperature to normal levels, whereas animals treated with colloidal solutions remained hypothermic (Table 1). The normalization of core temperature and the improvement of baseline physiological functions may be associated with enhanced oxygen delivery to tissues, thereby supporting cellular metabolism

HBOCs were more effective in restoring the systolic arterial pressure (SAP) pressure compared to other fluid therapies. This trend was observed across all study parameters, with diastolic arterial pressure (DAP) initially decreasing after shock induction, but subsequently returning to baseline following isovolemic HBOC transfusion. The most significant improvement was observed in the mean arterial pressure (MAP), which showed a substantial increase in the HBOC group compared to both the control and AT groups at later time points (T7 and T8). While MAP values in the HBOC and AT groups were similar, they were significantly higher in the group treated with colloidal (Col) solutions (Table 2).

### 3.2. Arterial Blood Gases Evolution

The evolution of measured arterial blood gases during the experimental procedure is presented in Table 3.

The normal pH in rabbits ranges approximately from 7.34 to 7.50 [21]. At T1, all groups exhibited pH values within normal physiological limits, although BE started slightly below reference values, indicating a mild subclinical metabolic imbalance not yet reflected in the pH. Following hemorrhagic shock induction, a decrease in pH was observed across groups, consistent with the development of metabolic acidosis, as evidenced by a further drop in BE and a slight elevation in pCO_2_—suggesting a mixed acidosis pattern. The pCO_2_ was far above the normal upper limit of 40 mmHg [22]. Although pCO_2_ decreased, it remained above the upper physiological limit. During the resuscitation phase, pH values normalized in both the colloid and HBOC groups, in parallel with a moderate improvement of BE, reflecting the correction of the metabolic acidosis. The HBOC group maintained a stable pH (7.38 ± 0.07) within physiological limits, exhibiting only a slight acidifying effect. The minor pH decrease observed from T6 to T8 in this group may be attributed to the metabolic processing of the hemoglobin-based oxygen carrier, which appears to have a less pronounced impact on systemic acid–base balance. In contrast, the AT group displayed marked alkalosis at T8, pH (*p* < 0.01) reaching 7.76 ± 0.15, which was accompanied by a substantial rise in BE (7.7).

Although statistically insignificant, pCO_2_ levels decreased significantly (*p* < 0.05) following the induction of hemorrhagic shock. Both the colloid and HBOC groups exhibited a continuous decreasing trend in pCO_2_, while the AT group showed an increase in pCO_2_ levels after reinfusion was completed.

Levels of the total CO_2_ in arterial blood were significantly altered following the induction of hemorrhagic shock, with an increasing trend initially observed, followed by a continuous decline after volume recovery in all animals.

Overall, HBOC treatment effectively preserved physiological pH throughout the experiment, demonstrating its ability to preserve the acid–base balance during hemorrhagic shock and post-transfusion, with a more controlled effect on blood pH compared to the alkalosis observed with conventional transfusion treatments.

### 3.3. Arterial Blood Hemoglobin Parameters

Oxyhemoglobin displayed a decreasing trend following the administration of HBOC as presented in Table 4. This trend is associated with changes in PaO_2_ and SO_2_ levels, which may be attributed to the high oxygen binding properties of the administered HBOC.

Initially, the hemoglobin and hematocrit levels were within normal ranges, and, expectedly, showed a significant decrease following the induction of hemorrhagic shock. In the colloid transfusion group, hemoglobin levels continued to decrease sharply, as colloid solutions act primarily as vascular expanders and lack hemoglobin molecules. In contrast, during autohemotransfusion, a marked restoration in hemoglobin levels was observed due to the reinfusion of the animal’s own blood. However, hemoglobin levels remained below the lower physiological reference value. The administration of HBOC had a similar effect to the colloid transfusion fluid. At T8, the AT group demonstrated an increase in hematocrit levels, suggesting the reintroduction of red blood cells (RBCs) through autotransfusion. Before the autotransfusion, the hematocrit level was 20.00 ± 1.41%, with levels registering an increase at 25.00 ± 1.41% after transfusion was finished. This aligns with the objective of autotransfusion, which restores RBC volume. In contrast, the HBOC group exhibited a continued decrease in hematocrit levels, starting from 24.00 ± 1.41% prior to the transfusional procedure and registering a value of 21.00% post-transfusion, confirming that HBOC solutions are not designed to restore RBC volume but rather to act as oxygen carriers.

### 3.4. Electrolyte Measurement in Arterial Blood

Various electrolytic measurements were performed during the experimental procedures presented in Table 5.

No significant changes were observed in the levels of ions such as sodium and potassium during the experimental procedures. However, notable alterations were seen in the progression of ionized calcium levels, which followed a continuous downward trend. After the transfusion procedures, ionized calcium levels continued to decrease. This decline was particularly evident in the AT group, likely due to the presence of anticoagulant sodium citrate in the transfusion bag, which acts as a calcium chelator agent

Mildly elevated glucose levels were likely a result of ischemic stress induced by hemorrhagic shock, compounded by the effects of prolonged anesthesia [23].

The statistically significant increase in the anion gap, despite largely unchanged electrolyte levels, can be explained by several factors. At baseline (T1), anion gap values were within normal ranges across all groups. By T6, an increase was noted, likely due to lactate accumulation during hemorrhagic shock. Although resuscitation led to a slight decrease, the values did not fully return to baseline levels. Notably, the HBOC group demonstrated a more pronounced rise in the anion gap, potentially attributable to the electronegative properties of hemoglobin-based oxygen carriers (HBOCs), which may contribute to the increase.

Other mechanisms related to HBOC metabolism may also contribute to the observed effects. The renal metabolism of HBOCs could lead to the accumulation of unmeasured anions in the plasma, thereby increasing the anion gap.

Ionized magnesium (iMg) levels displayed a similar trend across all experimental groups, while remaining within reference ranges. At the second time point, iMg levels increased compared to baseline, but decreased again by the third time point. In the AT group, iMg levels at the third time point (0.70 ± 0.01) were significantly higher than baseline values and differed significantly from the HBOC group at the same time point. The significant difference between the AT and HBOC groups at T8 may suggest that HBOCs are more effective in managing acidosis and cellular damage compared to whole blood transfusions. Chlorine levels showed no statistically significant changes throughout the experimental procedures.

### 3.5. Metabolic Products Measurement in Arterial Blood

As detailed in Table 6, creatinine and Blood Urea Nitrogen (BUN) levels remained within physiological ranges, suggesting that the tested hemoglobin-based oxygen carriers (HBOCs) may offer superior renal safety compared to previously developed HBOCs, which have demonstrated significant nephrotoxic effects. These earlier formulations required further investigation into the underlying mechanisms of their production.

Total bilirubin levels showed no statistically significant changes.

Following isovolemic transfusions, contrary to expectations, post-transfusion lactate levels were higher in the HBOC group. This trend may be attributed to the potential interference of HBOCs in plasma, which can affect the accuracy of lactate measurements, particularly at elevated concentrations.

### 3.6. Respiratory Gas Evolution During Experimental Procedures

No significant changes were observed in blood oxygen saturation over the course of this study. However, as presented in Table 7, notable alterations were seen in the measured end-tidal CO_2_ (ETCO_2_) levels. In conjunction with total CO_2_ (TotCO_2_), partial pressure of CO_2_ (pCO_2_), and arterial oxygen pressure (PaO_2_) values, ETCO_2_ was found to be associated with metabolic acidosis secondary to hemorrhagic shock. During resuscitation, a progressive increase in ETCO_2_ was noted, with final elevated levels potentially resulting from prolonged anesthesia.

## 4. Discussion

Regarding vitals and in consistency with our findings, a study by Williams et al. (2021) [24] suggested that PolybHb is as effective as whole fresh blood in improving cardiac function. Furthermore, these studies reaffirm that the PolybHb remains undamaged and safe even after extended storage [24,25,26]. A study by Day (2003) [27] concluded that HBOCs demonstrated comparable safety and efficacy to traditional resuscitation methods, including colloid and crystalloid solutions, in a hemorrhagic shock model. Notably, HBOC was particularly effective in restoring blood pressure and achieving normal mean arterial pressure (MAP) and systolic blood pressure levels more efficiently than other treatment groups.

The dramatic elevation in BE in the AT group could be attributed to the metabolism of citrate from CPDA-stored blood into bicarbonate, a known cause of post-transfusion metabolic alkalosis [28]. These findings highlight the differential metabolic impacts of the resuscitation fluids used in this study. As such, careful monitoring of these potential complications is crucial for patients receiving whole blood and blood products regularly.

The evolution of pCO_2_ was studied by Williams et al. (2014) [24] which demonstrated that the decreasing trend of pCO_2_ from baseline in hemorrhaging animals is indicative of poor tissue oxygenation and may serve as a more reliable marker of impending shock. Additionally, Gvalladhze et al. (2011) [29] suggested that the reduction in pCO_2_ observed during colloid administration could be attributed to CO_2_ consumption in the renal tubules for bicarbonate synthesis.

Kim et al. (2015) [30] demonstrated that the accuracy of total carbon dioxide measurement has been debated, as its reliability may vary depending on the study, equipment, or calibration method used. Therefore, total CO_2_ should be interpreted in accordance with institutional standards and in conjunction with other markers such as sodium bicarbonate levels and end-tidal CO_2_.

Considering HBOC high levels of oxygen binding, Day (2022) [27] demonstrated that when hemoglobin is extracted from red blood cells, the reduced levels of 2,3-diphosphoglycerate (2,3-DPG)—a molecule that plays a critical role in modulating the binding and release of oxygen by hemoglobin—result in a leftward shift in the oxyhemoglobin dissociation curve. This shift reduces the amount of oxygen released to tissues. In pure hemoglobin solutions, the partial pressure of oxygen required to achieve 50% hemoglobin saturation (P50) is approximately 10 mmHg. As a result, while these solutions can carry substantial amounts of oxygen, they are less efficient at delivering oxygen to tissues. The slight increase in carboxyhemoglobin levels observed throughout the experiment, although remaining within physiological limits, can likely be explained by the same mechanisms previously described.

Olofsson et al. (2008) [31] concluded that polymerized or conjugated hemoglobin, similar to other acellular HBOCs, lacks the ability to autoregulate the oxidative state of iron (Fe) within their heme groups. Consequently, Fe^2+^-containing hemoglobin is irreversibly converted into Fe^3+^-containing methemoglobin (metHb). Furthermore, Cabrales et al. [32] demonstrated that metHb exhibits a high affinity for oxygen but a low oxygen-carrying capacity, in contrast to normal hemoglobin, impairing oxygen delivery even at physiological levels. Supporting this concept, Elmer et al. (2012) [33] found that this conversion leads to detrimental effects, such as bradycardia and hypotension. In our study, while metHb levels remained statistically insignificant, they were maintained within physiological ranges throughout all experimental procedures, suggesting an adequate oxygen-carrying capacity without significant interference with the oxidative state of iron.

As hypocalcemia was observed in the AT group, a comprehensive study on the effects of transfused citrate in hemorrhagic shock [34] concluded that while the impact of citrate beyond hypocalcemia remains unclear, its delayed clearance in hemorrhagic shock exacerbates the deadly cycle of coagulopathy, acidemia, hypothermia, and hypocalcemia. This impairs clotting, increases transfusion requirements, and introduces more citrate into the system. Whole blood transfusions help to alleviate this burden, making it crucial to minimize citrate exposure and treat hypocalcemia [28,34]. Roghani et al. (2014) [35] highlighted that despite advancements in hemoglobin-based oxygen carriers (HBOCs), challenges such as coagulopathy, nitric oxide scavenging, platelet interference, and reduced calcium levels due to volume expansion remain. Ionized magnesium levels exhibited a similar trend across all experimental groups, maintaining values within reference ranges.

Anion gap evolution was detailed by a study investigating the effects of HBOC-201 in a swine model of severe hemorrhage and traumatic brain injury that noted that infusion was associated with low pH and a high anion gap, indicating anionic gap acidosis. This suggests that HBOC-201 may contribute to metabolic acidosis, potentially through mechanisms involving its biochemical properties [36].

Research on surface-modified hemoglobin microparticles supports this perspective, revealing that these particles possess a negative charge essential for their stability and function. While this characteristic is crucial for maintaining the functionality of HBOCs, it may indirectly influence systemic acid–base balance and contribute to the observed increase in the anion gap [37]. These properties stem from components such as phosphate, carbonate, ketone bodies, and lactic acid [38]. This interpretation emphasizes the need for further investigation into the role of HBOCs’ electronegativity in altering systemic acid–base dynamics and its clinical implications during resuscitation.

An increase of ionized magnesium during whole blood transfusion was noted in a study by Lee et al. (2017) [39] concluded that elevated ionized magnesium and total magnesium levels result from Mg^2+^ efflux from metabolically damaged cells during acidosis and ATP depletion.

The nephrotoxicity observed in these earlier HBOCs was attributed to the dissociation of hemoglobin tetramers into dimers. These dimers exhibit a high affinity for oxygen but lack cooperativity and allosteric regulation, leading to leakage into tissues, depletion of endothelial nitric oxide (NO), and consequently hypertension, as well as kidney toxicity [40,41]. Furthermore, Wang et al. (2014) [25] highlighted the promise of PEGylated hemoglobin (Hb) as a potential HBOC, noting its non-nephrotoxic properties.

Lactate, a critical marker of ischemic damage, demonstrated a marked increase from baseline following hemorrhagic shock, reflecting impaired tissue perfusion [16,42]. However, the interference with lactate measured concentrations arises because some types of HBOCs can absorb light at similar wavelengths used in lactate assays, leading to falsely elevated lactate levels [42].

In accordance with our observations, previous studies on HBOC present mechanistic explanations for the observed superiority of glutaraldehyde-polymerized ovine hemoglobin (PolyHb) compared to polymerized bovine or human hemoglobin. These differences refer to variations regarding potential enhanced biocompatibility and reduced toxicity. In our previous studies, ovine PolyHb exhibits superior performance in hemorrhagic shock models compared to bovine PolyHb. Specifically, ovine PolyHb demonstrated better survival rates and more favorable histopathological and immunological profiles, suggesting that ovine hemoglobin may induce less oxidative stress and inflammatory responses, with potential improved biocompatibility [2]. Ovine PolyHb may retain more favorable oxygen-binding characteristics post-polymerization, enhancing its efficacy as an oxygen carrier [43]. Furthermore, while both bovine and human PolyHb have been associated with vasoactivity, ovine PolyHb’s polymerization may result in a molecular structure that minimizes these adverse effects [29]. There is evidence supporting the notion that species-specific structural differences in hemoglobin influence the outcomes of glutaraldehyde polymerization, which may contribute to the observed superiority of ovine hemoglobin-based oxygen carriers (HBOCs) over those derived from bovine or human sources [44]. Finally, polymerized bovine hemoglobin has been shown to have increased rates of autoxidation and formation of ferryl hemoglobin, which can be detrimental. Ovine PolyHb may exhibit better redox stability, reducing the risk of oxidative damage during use [43].

As ETCO_2_ levels were markedly elevated during final volemic resuscitation periods, Gografe et al. (2003) [45] demonstrated this phenomenon in rabbits under long-term general anesthesia, where despite no oxygenation issues and no correlation between elevated perioperative ETCO_2_ and recovery time, over half of the animals had ETCO_2_ levels exceeding 45 mmHg at least once during the procedure. Furthermore, more than a quarter of the rabbits maintained elevated ETCO_2_ for over an hour. Additionally, Ghvalladhze et al. (2011) [46] suggested that the reduction in PCO_2_ observed during colloid administration could be attributed to CO_2_ consumption in the renal tubules for bicarbonate synthesis.

Hemoglobin-based oxygen carriers (HBOCs) are gaining attention in veterinary medicine as an innovative solution to persistent challenges like blood shortages, species-specific compatibility issues, and the complexities of storing blood in remote or resource-constrained environments. These carriers hold particular promise in critical care settings, including trauma, hemorrhagic shock, and surgical interventions, where efficient oxygen delivery can be life-saving. Cao et al. researched a 25-year review on HBOC applicability in veterinary medicine. They noted that in cases where RBCs are unavailable, HBOCs can provide critical oxygen support, ensuring tissue oxygenation until RBC levels recover sufficiently to sustain life [47]. Potential side effects, including vasoconstriction, elevated methemoglobin levels, and potential cardiac complications [48,49,50], which highlight the importance of careful monitoring, but can be managed with proper clinical intervention. HBOC-201 plays a critical role as a temporary oxygen carrier, effectively enhancing oxygenation at both local and systemic levels. Therefore, HBOC-201 serves as a practical alternative to resuscitation fluids and red blood cells (RBCs) in emergency situations. Some HBOC products such as Oxyglobin, similar to Hemopure [51] products, are also currently approved for veterinary use in the USA an EU [42,51]. Their versatility make them applicable across a range of conditions involving oxygen deprivation, such as traumatic hemorrhagic shock, hemolysis, myocardial infarction, and others. Unlike traditional resuscitation fluids, HBOC has the unique ability to bind oxygen and release it to tissues, functioning as both a volume expander and an oxygen carrier [47].

HBOCs have been extensively researched in human medicine, considered as ideal blood substitutes that could provide reliable oxygen transport, have minimal immunogenicity, demonstrate renal compatibility, and confer no risk of disease transmission, among other advantages. Additionally, HBOC should be easy to store, widely available, and scalable for mass production [52,53].

Efforts to enhance oxygen delivery through HBOCs have been hindered by toxicity concerns. Genetic and chemical modifications, such as polymerization and cross-linking, have improved their safety and efficacy by reducing the adverse effects of cell-free hemoglobin [53,54]. Another promising approach involves recombinant hemoglobin, which offers advantages like a natural origin, longer shelf-life, reduced disease transmission risk, and standardized production. However, optimizing recombinant Hb remains challenging, requiring specific mutations to minimize oxidation, heme loss, and nitric oxide scavenging while maintaining essential hemoglobin properties. This strategy holds potential as a sustainable and unlimited source of hemoglobin [53,55].

The ovine-derived HBOC tested in this study shows promising potential for managing hemorrhagic shock, with efficient oxygen-carrying capacity and a favorable safety profile. Its polymerized form may reduce vasoactivity, and the use of ovine hemoglobin offers logistical advantages, such as availability and low zoonotic risk. These features support its potential as an effective alternative to traditional transfusion, especially in emergency or resource-limited settings. However, further investigation into long-term effects, immunogenicity, and scalability in clinical use should be considered.

## 5. Conclusions

Hemoglobin-based oxygen carriers (HBOCs) are emerging as promising alternatives to traditional blood transfusions, offering substantial potential for oxygen delivery in various clinical settings. However, their use presents several challenges that must be addressed.

The studied ovine hemoglobin, polymerized with glutaraldehyde, exhibits a volume expansion capacity comparable to whole blood transfusions, with significantly superior efficacy in maintaining arterial blood pressure during hemorrhagic shock compared to synthetic colloid fluids like Dextran 40. Throughout the experiment, HBOC treatment effectively maintained physiological pH, highlighting its potential in managing acid–base balance during hemorrhagic shock and post-transfusion. This approach demonstrated a more controlled impact on blood acidification compared to standard transfusion protocols. Elevated CO_2_ levels were also noted in cases of respiratory acidosis, particularly when PaCO_2_ was elevated. However, total CO_2_ (TotCO_2_) can increase in cases of metabolic alkalosis, as reflected in the evolution of the end-tidal CO_2_ (ETCO_2_) curves.

HBOCs interact directly with blood components, such as bicarbonate (HCO_3_), potentially leading to a reduction in total CO_2_ due to the acidic nature of hemoglobin outside of red blood cells. Furthermore, HBOCs can interfere with lactate measurement, particularly at higher concentrations. As such, caution should be exercised when interpreting clinical markers, such as lactate and total CO_2_, in the presence of HBOC to avoid misdiagnosis or inappropriate therapeutic interventions. Trends in ionized magnesium suggest that HBOCs may be more effective than whole blood transfusions in controlling acidosis and cellular damage. Post-transfusion, ionized calcium levels showed a continuous decrease due to volume expansion. Creatinine and BUN levels remained within physiological ranges, suggesting that the HBOCs used in this study possess superior renal safety compared to earlier HBOC, which demonstrated significant nephrotoxic effects and necessitated further investigation into their production mechanisms.

HBOCs represent a promising solution for oxygen delivery, their effects on blood chemistry particularly, with CO_2_ and lactate levels needing to be carefully considered. Although no direct oxygenation issues were observed in the experimental models, the rise in CO_2_ levels and the interference of HBOCs with lactate measurements highlight the importance of thorough clinical monitoring. PEGylated hemoglobin offers a non-nephrotoxic alternative, yet challenges remain in preventing nitric oxide scavenging and ensuring effective oxygen delivery. Therefore, while HBOCs show significant potential, their use requires cautious interpretation of physiological markers and a thorough understanding of their interactions to avoid complications such as hypertension, acidosis, and organ damage.

## 6. Study Limitations

This study presents several limitations that must be acknowledged. First, the number of animals used was restricted by bioethical regulations and approval from the institutional ethics committee. This limitation, while essential for ensuring compliance with animal welfare standards, may reduce the statistical power and generalizability of the findings.

Second, this study focused exclusively on short-term effects, with a limited monitoring period. This design enabled the assessment of immediate hemodynamic and metabolic responses but did not allow for the evaluation of long-term outcomes, including potential delayed toxicities or adverse reactions.

Despite these limitations, this study provides important insights into the acute resuscitative potential of ovine HBOC in hemorrhagic shock and lays the groundwork for future studies with extended monitoring and broader comparative analysis. Further studies are warranted to explore long-term effects, comparative performance with other resuscitation products, and potential immunological responses in extended experimental models.

## Figures and Tables

**Table 1:** Heart rates, respiratory rates, and core body temperatures during experimental transfusion procedure.

Group	T1	T2	T3	T4	T5	T6	T7	T8
mHR (beats/min)								
Col	206.66 ± 5.73	142.66 ± 2.49	165.60 ± 4.10	170.00 ± 7.07	186.60 ± 16.11	174.60 ± 8.37	166.60 ± 23.58	176.30 ± 13.07
AT	190.33 ± 1.24	176.66 ± 4.98	177.00 ± 1.63	183.33 ± 4.78	171.60 ± 27.62	173.66 ± 10.27	188.33 ± 6.23	240.66 ± 26.44
HBOC	194.66 ± 2.35	200.00 ± 1.41	265.00 ± 35.35	174.00 ± 1.41	208.33 ± 25.30	206.00 ± 4.24	232.00 ± 14.23 *^,†^	239.00 ± 9.41 **
RR (breaths/min)								
Col	49.66 ± 6.84	41.00 ± 3.26	39.00 ± 2.16	38.33 ± 0.94	49.66 ± 4.49	46.66 ± 2.05	33.33 ± 5.43	48.33 ± 4.71
AT	31.66 ± 1.24	36.00 ± 1.63	39.00 ± 6.68	29.00 ± 2.16	38.66 ± 4.02	30.33 ± 1.24	31.33 ± 1.24	40.50 ± 3.5
HBOC	25.00 ± 6.48	24.00 ± 7.87	12.33 ± 3.68	25.33 ± 2.62	28.66 ± 6.12	36.66 ± 17.32	65.66 ± 14.29	55.66 ± 5.18
T (°C)								
Col	38.23 ± 0.12	38.20 ± 0.16	37.86 ± 0.26	37.53 ± 0.24	37.40 ± 0.14	37.36 ± 0.04	37.26 ± 0.32	37.20 ± 0.28
AT	38.96 ± 0.28	38.76 ± 0.12	38.13 ± 0.28	37.86 ± 0.36	37.76 ± 0.16	36.96 ± 0.09	38.03 ± 0.57	38.46 ± 0.36 **
HBOC	38.36 ± 0.68	38.46 ± 0.59	37.73 ± 0.24	38.06 ± 0.59	37.16 ± 0.61	37.20 ± 0.37	38.06 ± 0.47	38.36 ± 0.18 **

(mean ± SD, 5 animals/group) * *p* < 0.05, ** *p* < 0.01; compared to Col group, ^†^ *p* < 0.05 compared to AT group. mHR = heart rate; RR = respiratory rate. T = core body temperature. Reference range in HR = 112–300 (/min); RR = 30–60 (/min); T = 38.4–39.4 (°C), carotidian catheters placed (T1), blood withdrawal values every 15 min (T2, T3, T4), transfusion starting after 60 min of induced hemorrhagic shock (T5), post-transfusion 15 min (T6), 30 min (T7), 1 h (T8).

**Table 2:** Hemodynamic parameters during experimental transfusion procedure.

Group	T1	T2	T3	T4	T5	T6	T7	T8
SAP								
Col	109.33 ± 0.47	107.33 ± 1.69	84.00 ± 0.81	80.66 ± 1.24	70.33 ± 1.69	49.33 ± 4.92	55.66 ± 5.43	60.00 ± 10.23
AT	101.00 ± 6.97	86.33 ± 3.09	78.33 ± 3.09	60.66 ± 1.69	52.66 ± 1.69	50.00 ± 0.81	68.00 ± 1.63	65.00 ± 4.08
HBOC	94.66 ± 18.92	87.66 ± 3.85	74.66 ± 2.62	74.00 ± 16.26	66.00 ± 5.88	58.00 ± 9.09	80.33 ± 8.99 *	93.66 ± 4.49 *
DAP								
Col	84.33 ± 1.69	80.66 ± 0.47	64.00 ± 0.81	61.00 ± 1.63	58.33 ± 0.94	43.00 ± 1.63	54.66 ± 5.52	55.33 ± 11.61
AT	86.00 ± 0.81	76.00 ± 2.16	71.00 ± 2.94	55.00 ± 2.16	48.66 ± 0.47	45.33 ± 1.24	48.00 ± 8.83	48.66 ± 10.94
HBOC	89.33 ± 10.33	78.00 ± 12.02	69.00 ± 5.09	61.33 ± 2.49	50.33 ± 6.18	41.00 ± 1.41	61.00 ± 4.16	71.33 ± 11.48 *
MAP								
Col	92.33 ± 1.24	89.00 ± 0.81	71.00 ± 0.81	67.33 ± 2.05	62.66 ± 1.24	45.66 ± 2.05	54.00 ± 5.71	57.66 ± 5.55
AT	91.00 ± 0.81	79.66 ± 2.05	73.33 ± 1.69	57.00 ± 0.81	49.33 ± 1.24	47.00 ± 0.81	55.00 ± 5.71	54.00 ± 10.19
HBOC	91.66 ± 13.29	81.00 ± 9.20	71.66 ± 0.47	65.66 ± 13.22	55.00 ± 4.54	47.00 ± 2.16	67.33 ± 5.83	78.00 ± 19.20 *

SAP = systolic arterial pressure. DAP = diastolic arterial pressure. MAP = mean arterial pressure. Reference range in SAP = 90–130 mmHg. (mean ± SD, 5 animals/group) * *p* < 0.05; compared to Col group. Reference range DAP = 80–90 mmHg. Reference range MAP = 83–103 mmHg, carotid catheters placed (T1), blood withdrawal values every 15 min (T2, T3, T4), transfusion starting after 60 min of induced hemorrhagic shock (T5), post-transfusion 15 min (T6), 30 min (T7), 1 h (T8).

**Table 3:** Arterial blood gases measured at various time points during experimental HBOC transfusion procedure study.

Group	T1	T6	T8
pH			
Col	7.33 ± 0.14	7.12 ± 0.05	7.36 ± 0.14
AT	7.35 ± 0.03	7.15 ± 0.07	7.76 ± 0.15 **
HBOC	7.37 ± 0.04	7.11 ± 0.10	7.38 ± 0.07 ^††^
PCO_2_ (mmHg)			
Col	56.30 ± 40.87	48.45 ± 10.82 *	43.05 ± 36.84
AT	54.75 ± 13.51	44.35 ± 12.23 *	47.70 ± 88.67
HBOC	50.85 ± 18.88	43.00 ± 14.04	40.55 ± 27.37
PO_2_ (mmHg)			
Col	98.20 ± 51.48	196.60 ± 39.03	112.25 ± 176.85
AT	100.25 ± 11.21	174.35 ± 114.76	110.75 ± 48.86
HBOC	95.65 ± 1.20 *	167.75 ± 69.93	104.05 ± 3.45
SO_2_ (%)			
Col	99.50 ± 3.54	99.50 ± 0.61	91.00 ± 1.41
AT	100.00 ± 0.10	99.50 ± 0.33	92.00 ± 1.41
HBOC	99.50 ± 0.71	99.50 ± 0.71	99.50 ± 0.71 ^††^
TotCO_2_ (mmol/L)			
Col	30.05 ± 7.71	49.40 ± 0.99	29.15 ± 5.40
AT	31.40 ± 5.52	48.90 ± 3.96	38.85 ± 0.92 **
HBOC	28.50 ± 4.42	46.20 ± 2.06	19.55 ± 3.65 ^††^
BE ecf (mmol/L)			
Col	3.65 ± 1.17	−7.80 ± 0.14	1.25 ± 6.01
AT	3.00 ± 3.68	−8.90 ± 0.57	7.70 ± 0.99 **
HBOC	2.05 ± 4.35	−9.55 ± 3.12	−1.90 ± 11.74 ^††^

(mean ± SD, 5 animals/group) * *p* < 0.05, ** *p* < 0.01; compared to Col group; ^††^ *p* < 0.01 compared to AT group. pCO_2_ = partial carbon dioxide pressure in arterial blood. PO_2_ = partial oxygen pressure in arterial blood. SO_2_ = oxygen saturation in arterial blood. TotCO_2_ = total carbon dioxide in arterial blood. BE ecf = base deficit in arterial blood. Reference range Ph = 7.24–7.53. Reference range PO_2_ = 75–101 mmHg. Reference range pCO_2_ = 28.9–58.2 mmHg. Reference range SO_2_ = 90–100%. Reference range TotCO2 = 18–34. Reference range BE ecf = −5.00–(6.00). Values were measured before hemorrhagic shock (T1), 30 min after the shock was induced (T6), and 60 min after isovolemic transfusions (T8).

**Table 4:** Hemoglobin parameters measured from arterial blood at various time points during an experimental HBOC transfusion procedure study in New Zealand white rabbits.

Group	T1	T6	T8
O_2_Hb (%)			
Col	94.23 ± 3.10	98.43 ± 1.08	92.63 ± 0.61
AT	99.36 ± 0.12	98.23 ± 0.80	93.30 ± 0.61
HBOC	99.16 ± 0.44	97.73 ± 0.38	97.93 ± 0.23 ^††^
COHb (%)			
Col	0.93 ± 0.68	0.96 ± 0.87	1.66 ± 0.88
AT	0.26 ± 0.04	0.50 ± 0.08	1.13 ± 0.12
HBOC	0.30 ± 0.08	0.63 ± 0.04	1.10 ± 0.08
MetHb (%)			
Col	0.36 ± 0.04	0.33 ± 0.04	0.43 ± 0.04
AT	0.26 ± 0.04	0.20 ± 278 × 10^−17^	0.46 ± 0.04
HBOC	0.33 ± 0.04	0.30 ± 0.14	0.43 ± 0.12
Hgb (g/dL)			
Col	11.50 ± 0.99	6.95 ± 0.35	5.40 ± 1.13
AT	11.05 ± 1.48	6.80 ± 2.26	8.20 ± 1.84 **
HBOC	11.25 ± 0.92	6.15 ± 0.21	6.00 ± 0.28 ^††^
HCT (%)			
Col	35.00 ± 1.41	25.00 ± 1.41	17.00 ± 5.66
AT	34.00 ± 4.24	20.00 ± 1.41	25.00 ± 1.41 **
HBOC	34.50 ± 2.12	24.00 ± 1.41	21.00 ± 0.00 ^††^

(mean ± SD, 5 animals/group); ** *p* < 0.01; compared to Col group; ^††^ *p* < 0.01 compared to AT group. O_2_Hb = uxyhemoglobin. COHb = carboxyhemoglobin. MetHb = methemoglobin. Hgb = hemoglobin level. HCT = hematocrit. Reference range O_2_Hb = 91–99 mmol/L. Reference range COHb = 0.5–5%. Reference range pCO_2_ = 28.9–58.2 mmHg. Reference range MetHgb = 0.06–2.2%. Reference range Hgb = 10.7–13.9 mg/dL. Reference range HCT = 28–48. Values were measured before hemorrhagic shock (T1), 30 min after the shock was induced (T6), and 60 min after isovolemic transfusions (T8).

**Table 5:** Electrolytes measured from arterial blood at various time points during the experimental HBOC transfusion procedure study.

Group	T1	T6	T8
Na (mmol/L)			
Col	146.10 ± 5.66	143.90 ± 1.27	143.40 ± 3.39
AT	143.55 ± 2.76	143.80 ± 2.83	147.10 ± 6.51
HBOC	138.80 ± 2.83	138.25 ± 4.45	139.85 ± 5.30
K (mmol/L)			
Col	3.53 ± 0.42	4.18 ± 0.80	3.97 ± 0.30
AT	3.95 ± 0.33	3.96 ± 0.04	4.69 ± 0.79
HBOC	3.80 ± 0.15	4.24 ± 0.23	4.29 ± 0.08
iCa (mmol/L)			
Col	1.62 ± 0.04	1.54 ± 0.14	1.43 ± 0.13
AT	1.63 ± 0.14	1.55 ± 0.02	1.40 ± 0.02
HBOC	1.59 ± 0.01	1.33 ± 0.04	1.22 ± 0.00 ^††^
iMg (mmol/L)			
Col	0.61 ± 0.03	0.64 ± 0.13	0.60 ± 0.01
AT	0.61 ± 0.01	0.73 ± 0.02	0.70 ± 0.01 **
HBOC	0.70 ± 0.04	0.81 ± 0.08	0.65 ± 0.17
Glucose (mg/dL)			
Col	166.00 ± 80.61	167.50 ± 45.96	147.50 ± 79.9
AT	155.00 ± 26.87	153.00 ± 15.56	193.00 ± 2.83
HBOC	167.00 ± 93.34	164.00 ± 18.38	135.50 ± 33.23
Cl (mmol/L)			
Col	105.05 ± 0.21	104.90 ± 0.85	107.60 ± 1.41
AT	107.45 ± 8.13	106.20 ± 0.71	110.20 ± 0.42
HBOC	105.45 ± 5.44	103.90 ± 4.38	105.55 ± 2.19
AnGap (mmol/L)			
Col	11.25 ± 1.34	26.45 ± 2.33	13.22 ± 5.87
AT	11.50 ± 1.56	25.05 ± 1.34	14.15 ± 2.90
HBOC	12.50 ± 5.37	26.70 ± 12.30	20.35 ± 5.16 ^††,^**

(mean ± SD, 5 animals/group) ** *p* < 0.01 compared to Col group); ^††^ *p* < 0.01 compared to AT group. Na = sodium, K = potassium, iCa = ionized calcium, iMg = ionized magnesium. Cl = chlorine. AnGap = anion gap. Reference range Na = 136–147. Reference range K = 3.4–5.7. Reference range Cl = 93–113. Reference range iCa = 1.20–1.95. Reference range iMg = 0.6–1.05. Reference range Glucose = 75–150 mg/dL. Reference range AnGap = 11–26. Values were measured before hemorrhagic shock (T1), 30 min after the shock was induced (T6), and 60 min after isovolemic transfusions (T8).

**Table 6:** Metabolic products measured from arterial blood at various time points during experimental HBOC transfusion procedure study.

Group	T1	T6	T8
Creatinine (mg/dL)			
Col	0.90 ± 0.28	1.20 ± 0.28	1.05 ± 0.07
AT	0.90 ± 0.14	0.90 ± 0.28	0.85 ± 0.07 **
HBOC	1.00 ± 0.07	1.20 ± 0.07	1.25 ± 0.21 ^†^
BUN (mg/dL)			
Col	22.50 ± 0.71	25.00 ± 4.24	25.50 ± 3.54
AT	24.00 ± 1.41	24.50 ± 0.71	28.50 ± 0.71
HBOC	24.00 ± 0	25.00 ± 1.41	27.00 ± 7.07
Tot Bilirubin			
Col	1.20 ± 0.99	0.95 ± 0.49	0.50 ± 0.49
AT	0.65 ± 0.21	0.50 ± 0.14	0.85 ± 0.07
HBOC	0.95 ± 0.64	0.80 ± 0.42	1.10 ± 0.57
Lactate (mmol/L)			
Col	0.90 ± 0.07	2.20 ± 1.56	2.05 ± 0.07
AT	1.00 ± 0.28	2.19 ± 0.57	2.15 ± 0.35
HBOC	0.70 ± 0.28	2.15 ± 0.49	3.00 ± 1.27 **

(mean ± SD, 5 animals/group) ** *p* < 0.01 compared to Col group; ^†^ *p* < 0.05 compared to AT group. BUN = Blood Urea Nitrogen. TBilirubin = total bilirubin. Reference range creatinine 0.5–2.6. Reference range BUN = 9–33. Reference range T Bilirubin = 1–8. Reference range lactate = 0.9–1.7. Values were measured before hemorrhagic shock (T1), 30 min after the shock was induced (T6), and 60 min after isovolumic transfusions (T8).

**Table 7:** Respiratory gases measured at various time points during experimental HBOC transfusion procedure study.

Group	T1	T2	T3	T4	T5	T6	T7	T8
SO_2_%								
Col	99.66 ± 1.24	100.00 ± 0.00	99.33 ± 0.94	99.33 ± 0.47	99.33 ± 0.47	98.33 ± 0.47	98.00 ± 2.82	98.00 ± 2.82
AT	99.00 ± 0.81	99.66 ± 1.24	98.00 ± 0.81	99.66 ± 0.47	98.33 ± 1.69	99.33 ± 0.47	98.00 ± 0.81	98.00 ± 4.32
HBOC	99.00 ± 0.00	99.66 ± 0.47	99.33 ± 0.47	99.66 ± 0.47	99.33 ± 0.47	96.00 ± 2.82	99.00 ± 0.81	98.66 ± 1.24
ETCO_2_								
Col	31.33 ± 6.37	35.00 ± 0.47	30.33 ± 3.74	26.00 ± 2.62	26.33 ± 2.16	26.00 ± 2.62	38.33 ± 2.05	49.66 ± 9.53
AT	35.33 ± 6.62	32.33 ± 4.02	28.33 ± 2.05	24.66 ± 4.49	25.66 ± 3.39	26.33 ± 4.49	34.66 ± 1.69	52.66 ± 0.94
HBOC	34.33 ± 19.15	37.66 ± 3/39	30.33 ± 8.73	27.66 ± 8.04	26.00 ± 9.28	27.66 ± 3.69	34.33 ± 5.88	49.00 ± 4.54

(mean ± SD, 5 animals/group) SO_2_% = oxygen saturation. ETCO_2_ = end-tidal carbon dioxide in expiratory air. Reference range in SO_2_% = 96–100%. Reference range ETCO_2_ = 35–45 mmHg. Carotid catheters placed (T1), blood withdrawal values every 15 min (T2, T3, T4), transfusion starting after 60 min of induced hemorrhagic shock (T5), post-transfusion 15 min (T6), 30 min (T7), 1 h (T8).

## Data Availability

The original contributions presented in this study are included in the article. Further inquiries can be directed to the corresponding author(s).
